# Protein Carbonylation: Emerging Roles in Plant Redox Biology and Future Prospects

**DOI:** 10.3390/plants10071451

**Published:** 2021-07-15

**Authors:** Adesola J. Tola, Amal Jaballi, Tagnon D. Missihoun

**Affiliations:** Groupe de Recherche en Biologie Végétale (GRBV), Department of Chemistry, Biochemistry and Physics, Université du Québec à Trois-Rivières, 3351 boul. des Forges, Trois-Rivières, QC G9A 5H7, Canada; Adesola.Tola@uqtr.ca (A.J.T.); Amal.Jaballi@uqtr.ca (A.J.)

**Keywords:** protein carbonylation, signal transduction, reactive oxygen species, redox biology, proteasome-mediated degradation, proteome remodeling

## Abstract

Plants are sessile in nature and they perceive and react to environmental stresses such as abiotic and biotic factors. These induce a change in the cellular homeostasis of reactive oxygen species (ROS). ROS are known to react with cellular components, including DNA, lipids, and proteins, and to interfere with hormone signaling via several post-translational modifications (PTMs). Protein carbonylation (PC) is a non-enzymatic and irreversible PTM induced by ROS. The non-enzymatic feature of the carbonylation reaction has slowed the efforts to identify functions regulated by PC in plants. Yet, in prokaryotic and animal cells, studies have shown the relevance of protein carbonylation as a signal transduction mechanism in physiological processes including hydrogen peroxide sensing, cell proliferation and survival, ferroptosis, and antioxidant response. In this review, we provide a detailed update on the most recent findings pertaining to the role of PC and its implications in various physiological processes in plants. By leveraging the progress made in bacteria and animals, we highlight the main challenges in studying the impacts of carbonylation on protein functions in vivo and the knowledge gap in plants. Inspired by the success stories in animal sciences, we then suggest a few approaches that could be undertaken to overcome these challenges in plant research. Overall, this review describes the state of protein carbonylation research in plants and proposes new research avenues on the link between protein carbonylation and plant redox biology.

## 1. Introduction

Plants are sessile in nature and they perceive and react to the abiotic and biotic factors prevailing in their growth environment. Mechanistically, most environmental factors, such as high or low temperature, high light, drought, UV/X-ray irradiation, soil salinity, and nutrient deficiencies, induce a change in the cellular homeostasis of reactive oxygen species (ROS). The most reactive ROS include hydrogen peroxide (H_2_O_2_), the superoxide radical anion (O_2_^•−^), singlet oxygen (^1^O_2_), and the hydroxyl radical (HO^•^). ROS have different, short half-lives and they are endogenously produced during aerobic metabolism in plants. However, under stressful conditions, plants generate an enormous amount of ROS in their chloroplasts, peroxisomes, mitochondria and apoplasts. Although the enzymes that generate or process the ROS are well-known and found in nearly all taxa, how ROS encode the message from the environment is still elusive in plants. High intracellular levels of ROS are known to trigger the oxidation of diverse cellular components, including lipids, DNA, and proteins, and to contribute to cell and organismal ageing and death. Research findings from the last two decades have also shown that ROS act as signal molecules that control the expression of several plant genes involved in growth and development [1]. So far, well-known ROS signaling mechanisms in plants involve the direct oxidation of key cysteine residues in target proteins. ROS-mediated modification of proteins occurs through various post-translational modifications (PTMs), including the formation of intra- or intermolecular disulphide bonds between two cysteine residues (S-sulfhydration, glutathionylation), or one cysteine and nitric oxide or hydrogen sulfide (S-nitrosylation, persulfidation), and the oxidation reaction of H_2_O_2_ with the cysteine thiolate anion, leading to the formation of cysteine sulfenic acid (−SOH), sulfinic acid (−SO_2_H), and sulfonic acid (−SO_3_H) [2,3,4]. These modifications may activate or deactivate the target proteins or lead to their release from an interacting protein partner. ROS have also appeared to achieve their biological functions through the carbonylation of certain proteins [2]. In this case, ROS were shown to introduce carbonyl groups into the side chains of Lys, Pro, and Thr via the Fenton reaction or prompt the peroxidation of membrane lipids, which generate α,β-unsaturated aldehydes. These lipid-derived aldehydes then form carbonyl adducts on the side chains of Cys, His, and Lys in proteins in a non-enzymatic process [5]. Carbonylated proteins have been found at all the stages of the plant life cycle. In comparison to the animal models and to other ROS-mediated PTMs, little is known about their role in the regulation of gene expression and during plant growth and development [5,6]. Exogenous application of α,β-unsaturated aldehydes was found to trigger the expression of several genes implicated in growth and defense response in humans, animal models, and plants [7,8,9,10]; furthermore, a few studies have pointed to the role of protein carbonylation in diverse plant physiological processes [11,12,13,14]. Many proteins were carbonylated in *Arabidopsis thaliana* seeds during germination but these seeds successfully grew into highly vigorous seedlings and young plants, thus indicating a role for protein carbonylation in seed dormancy release and germination [11]. Like protein ubiquitinylation, carbonylation triggers proteasome-mediated degradation of proteins [15]. However, unlike protein ubiquitinylation or phosphorylation, protein carbonylation cannot be undone. Although recent genetic studies pointed to the transduction of ROS and phytohormone signals by protein carbonylation [16,17,18,19], it is still unclear how cells would control protein carbonylation in time and space similarly to ubiquitinylation or phosphorylation. The non-enzymatic feature of the carbonylation reaction and the fact that various endogenous lipid peroxidation-derived molecules can form adducts on proteins (see below) have considerably slowed the efforts to identify the functions regulated by protein carbonylation in plants.

In this review, we first describe the most common types of stress-derived ROS, the sites of their production in the cell, and the various PTMs that they trigger. Second, we focus on ROS-mediated protein carbonylation and the biological effects of this in selected prokaryotes and eukaryotes species. We discuss key aspects that need more attention in future investigations in plant species. These include the potential role of protein carbonylation in seed development and germination, protein quality control and proteome homeostasis, the crosstalk with other ROS-mediated protein modifications, hormone signaling, and the specificity of carbonylation targets. Finally, one important challenge in protein carbonylation research is the detection of carbonylated proteins by mass spectrometry. Based on examples of successful characterization of carbonyl adducts and their biological effects in bacteria and human cells, we present a few strategies that could also be implemented to bring progress in the field of plant research.

## 2. ROS: Diversity, Reactivity, and Sites of Production in Plants

**Figure 1:**
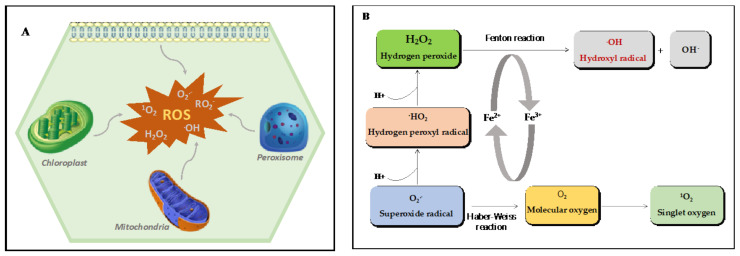
Environmental stress and ROS production in plants. (**A**) ROS are produced in the chloroplasts, mitochondria, peroxisomes, plasma membrane, cell walls, and apoplasts under normal growth conditions. However, environmental stresses exacerbate their production and may lead to oxidative stress. (**B**) The Fenton reaction and Haber–Weiss reaction. ROS are partially reduced or activated forms of molecular oxygen. About 1–5% of molecular oxygen consumed by plants leads to ROS generation. The different forms of ROS include superoxide radicals, singlet oxygen, hydrogen peroxide, perhydroxyl, and hydroxyl radicals.

ROS are partially reduced or excited forms of atmospheric oxygen with various levels of reactivity (Figure 1A,B) [20]. They are inevitable products of aerobic metabolism generated in the mitochondria of mammalian cells and non-photosynthesizing plant cells (Figure 1A). However, the main sources of ROS in photosynthesizing plant cells are the peroxisomes and the chloroplasts [21]. Diverse species of ROS can be derived from the transfer of sufficient energy capable of reversing the spin on unpaired electrons and thus producing ^1^O_2_ or during a sequential single-electron reduction pathway of molecular oxygen to produce O_2_^•−^, H_2_O_2_ or HO^•^ [22]. Under stress conditions, such as pathogen attacks, diseases, toxicity, and nutrient deficiencies, ROS production generally plummets and leads to oxidative modifications of macromolecules such as carbohydrates, unsaturated fatty acids, DNA, and proteins. Despite their potential toxicity, ROS also regulate several biological processes [21,23,24,25]. The most reactive and studied ROS in the biological systems include O_2_^•−^, H_2_O_2_, HO^•^, and ^1^O_2_.

### 2.1. ROS Diversity and Reactivity

#### 2.1.1. Singlet oxygen (^1^O_2_)

Singlet oxygen is non-radical molecular oxygen that possesses one unpaired electron in the π* orbital as a result of rapid decay of the free radical oxygen state with two unpaired electron states (non-radical) [22]. ^1^O_2_ is a short-lived molecule with a half-life of about 1–4 µs and can diffuse within a small diffusion range of 30 nm. It can potentially cause damage to lipids and proteins and induce cell death [26,27]. In plants, singlet oxygen is largely produced in the photosystem II (PS II) reaction center by photodynamic activation of the ground state oxygen that reacts with triplet chlorophyll [28]. Chloroplasts are thus the main site for ^1^O_2_ production and several carotenoid-dependent quenching systems can minimize the production of singlet oxygen, which dissipates as heat from excess light energy [29]. When carotenoid-dependent quenching becomes insufficient, excess ^1^O_2_ further triggers lipid peroxidation, which is essentially the main cause of stress-induced damage [27]. Despite its toxicity, singlet oxygen signaling is one of the important regulatory mechanisms of cell fate and gene expression [30,31].

#### 2.1.2. Superoxide Anion (O_2_^•−^)

There are various means through which superoxides are formed in a living system. The superoxide anion (O_2_^•−^) can be generated as a byproduct of aerobic metabolism during the sequential single-electron reduction pathway of molecular oxygen. Enzymes like xanthine oxidase and NAD(P)H oxidase carry out an electron reduction of molecular oxygen to form superoxide [32]. Since O_2_^•−^ has moderate reactivity and a short half-life (1–4 µs) [33,34], it is less aggressive than singlet oxygen and can further be reduced to H_2_O_2_ through singlet valency reduction or dismutation by superoxide dismutases (SODs).

#### 2.1.3. Hydrogen Peroxide (H_2_O_2_)

H_2_O_2_ lives longer than superoxide and has a half-life of about 1000 µs or more [35]. In plants, H_2_O_2_ is known to be a double-edged sword. At low cellular concentrations, it controls several vital physiological processes, whereas, at a high concentrations, it becomes harmful by causing oxidative damages to DNA and proteins [36].

#### 2.1.4. Hydroxyl Radical (HO^•^)

The hydroxyl radical is small, water-soluble, highly mobile, and well-known as the most reactive species of activated oxygen. It has a single unpaired electron with the shortest half-life of 1 µs [37,38] and, as a consequence, it is quick to irreversibly modify DNA, RNA, lipids, and proteins [39,40,41]. Hydroxyl radicals are formed from the non-enzymatic reaction of H_2_O_2_ in a process called the Fenton reaction (Figure 1A), where reduced metals, including Cu^2+^ or Fe^2+^, provide the electron to reduce H_2_O_2_ to the hydroxyl radical. There are no efficient enzymatic systems to scavenge toxic HO^•^ and excessive accumulation promotes programmed cell death [42]. The steady-state level of HO^•^ in the cell likely depends on the availability of H_2_O_2_ and metal ions and the rate of occurrence of the Fenton reaction and the Haber–Weiss reaction. Iron plays a significant role in the Fenton reaction in biological systems and is mostly present in its oxidized form (Fe^3+^). Only a small fraction of the iron pool exists in the oxidized form (Fe^2+^) and participates in the Fenton reaction. The Fenton reaction may be further enhanced by the Haber–Weiss reaction which generates Fe^2+^ from Fe^3+^ stored in ferritins [43] (Figure 1B).

### 2.2. ROS Production Sites in Plants

Studies have shown that an estimated 1–2% of oxygen consumption in plants results in the synthesis of ROS in different subcellular organelles, including mitochondria [44], chloroplasts [45], peroxisomes [46], apoplasts, and other possible compartments containing proteins and/or molecules with high redox potential as ROS synthetic sites (Figure 1A) [47,48].

#### 2.2.1. Chloroplasts

Chloroplasts are the major sites for ROS production in plants under illumination [45]. During photosynthesis, O_2_^•−^ and H_2_O_2_ are produced via the PS I [49] and the ^1^O_2_ in the PS II [50]. Singlet oxygen (^1^O_2_) is generated constitutively in illuminated chloroplasts and its synthesis is importantly enhanced under high or excess light or heat and short or suboptimal temperature stress conditions, which also limit CO_2_ fixation [51,52]. This results in the overloading of the electron transport chain (ETC), which eventually causes the leakage of an electron from ferredoxin to molecular oxygen to form superoxides (O_2_^•−^) and damaging the Fe-S cluster in the PSI [53]. This process is known as PSI photo-inhibition or the Mehler reaction. Superoxide dismutases present in the chloroplast convert O_2_^•−^ to H_2_O_2_ while ascorbate peroxidase and glutathione (GSH) peroxidase scavenge H_2_O_2_ back to water [54,55]. The Mehler reaction is thus integrated into the so-called water–water cycle, which may further enhance O_2_^•−^ production to H_2_O_2_ production.

#### 2.2.2. Mitochondria

In plants, ROS generation also takes place in the respiratory chain of the mitochondria at complex I (NADH dehydrogenase) and complex III (cytochrome b/c1 complex), which harbor electrons with adequate energy to reduce molecular oxygen to O_2_^•−^ [44,56]. O_2_^•−^ is produced from the single electron leak from complex I and III onto O_2_ in the ubiquinone. O_2_^•−^ is then scavenged by matrix-localized manganese superoxide dismutase to H_2_O_2_ and O_2_ [57]. About 1–5% of the oxygen used in the mitochondria results in the production of H_2_O_2_ that may subsequently be converted to HO^•^ via the Fenton reaction [58]. Under the normal respiratory conditions, ROS produced by chloroplasts and peroxisomes are more important than those produced in mitochondria. However, under stress conditions, ROS production in mitochondria is significantly enhanced and may lead to the activation of programmed cell death [59].

#### 2.2.3. Peroxisomes

Peroxisomes represent an additional site for O_2_^•−^ and H_2_O_2_ production apart from the chloroplasts and mitochondria. O_2_^•−^ are generated in the peroxisome matrix during the oxidation of xanthine to hypoxanthine and uric acid by xanthine oxidase or in the peroxisomal membrane during fatty acid beta-oxidation [46] and re-oxidation of NADH to yield NAD+ [60]. The concentration of H_2_O_2_ is two times higher in the peroxisomes than in chloroplasts and 50 times higher than in the mitochondria, partly due to photorespiration [61]. H_2_O_2_ is primarily produced in the peroxisomes by glycolate oxidase that catalyzes the oxidation of glycolate to glyoxylate in green tissues with the use of O_2_ as an electron donor [62,63]. Fatty acid β-oxidation, dismutation of O_2_^•−^ radicals, and enzymatic reactions catalyzed by flavin oxidases are other sources of H_2_O_2_ in the peroxisomes [64].

#### 2.2.4. Apoplasts (Plasma Membrane and the Cell Wall)

ROS are also produced in the apoplast, the compartment between the plasma membrane and the cell wall. The plasma membrane-localized NADPH oxidases are a major source of O_2_^•−^. Plant NADPH oxidases are homologs of mammalian respiratory burst oxidase homologs (RBOHs), and they possess an apoplastic oxidase domain that produces O_2_^•−^ in the apoplast [65]. O_2_^•−^ generated in the apoplast is subsequently converted to H_2_O_2_ by plasma membrane-localized superoxide dismutase [66]. Besides NADPH oxidase apoplasts, ROS are also produced through some enzymes present in the plant cell wall, including class III peroxidases, amine oxidases, lipoxygenases, and quinone reductase [67].

## 3. Common ROS-Mediated Post-Translational Modification (PTMs)

Despite their potential toxicity, ROS fulfill numerous biological functions in plants, mostly by modifying lipids and proteins. In this section, we describe the most common ROS-mediated post-translational modifications and protein carbonylation.

**Figure 2:**
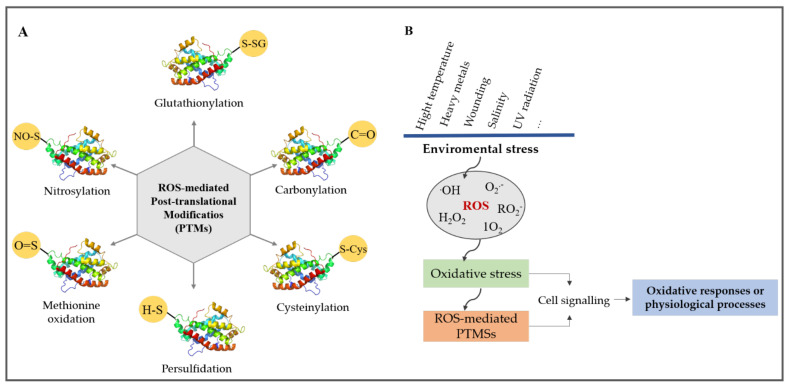
Overview of ROS-mediated post-translational modifications. (**A**) Most common ROS-mediated PTMs. The thiol groups of Cys and Met are prominently modified by ROS. The modifications include nitrosylation, cysteinylation, glutathionylation, methionine oxidation, persulfidation, and protein carbonylation. (**B**) Framework model of ROS-mediated PTMs in cell signaling in response to environmental stress in plants.

ROS can introduce covalent bonds into proteins by directly oxidizing amino acids (Cys residues) or create carbonyl groups in the side chains of certain amino acids [68,69,70]. The latter is termed protein carbonylation (Figure 2). Direct modification of proteins by ROS include nitrosylation or nitration of tyrosine residues, carbonylation of specific amino acids, formation of disulphide crosslinks, glycoxidation adducts, and glutathionylation, whereas indirect modification of a protein by ROS is prominently due to the conjugation of proteins to the breakdown products of lipid peroxidation [71] (Figure 2A). The accumulation of ROS in biological systems can result in either of these modifications, and they can be reversible or irreversible, interconnected, and influence each other [72] (Figure 2B).

### 3.1. Methionine Oxidation

Methionine oxidation is a reversible modification that occurs when the sulfur atom of methionine is oxidized to sulfoxide. Small heat shock proteins found in the chloroplasts are inactivated by sulfoxidation of methionine but reactivated by its reduction. The reduction is catalyzed by peptide methionine sulfoxide reductase using thioredoxin as a reductant [73]. Reports have suggested that methionine residues found in some peripherals act as endogenous antioxidants, protecting the active site and other sensitive domains in the protein while quenching ROS [74]. Reversible methionine sulfoxidation is deemed an important regulatory mechanism [75]. Further oxidation of methionine to sulfonic is irreversible and damaging to proteins [76].

### 3.2. Cysteinylation (Cysteine Oxidation) and Glutathionylation

Cysteine is readily oxidized by ^1^O_2_ and HO^•^ to form a disulfide bond (R1-S-S-R2, cystine) and this represents an important regulatory mechanism of the metabolism [77]. Initial oxidation of Cys by H_2_O_2_ typically leads to sulfenic acid, which can then form a mixed disulfide bond with GSH or a disulfide bond with other thiols. Further oxidation of sulfenic acid results in the formation of sulfinic acid and sulfonic acid. The sulfinic acid group may be reduced by a sulfiredoxin enzyme in mitochondria or peroxiredoxins in the chloroplast [78]. Glutathionylation involves the transfer of GSH, a tripeptide (Glu-Cys-Gly) available in plant cells in millimolar concentrations, to thiol groups in proteins. GSH can also form a mixed disulphide bridge with an accessible free thiol on a protein, a reaction termed protein S-glutathionylation. Several plant enzymes, typically proteins in the mitochondria, chloroplast, and the cytosol, have been proved to undergo glutathionylation [79].

### 3.3. Nitrosylation

This modification involves the covalent attachment of a nitrosyl radical (NO^•^) to a cysteine thiol group. NO is a lipophilic gas produced during abiotic stress and is recognized as an important regulator and signaling molecule in plants [80,81,82,83]. The thiol group in proteins (and glutathione) interacts with NO^•^ derivatives and results in a range of products, including disulfides and sulfenic, sulfinic, and sulfonic acids, as well as S-nitrosothiols [84]. The most well-known NO^•−^ derivative is peroxynitrite (ONOO^−^), which is a product of the condensation reaction of NO^•^ with O_2_^•−^. However, NO^•^ does not cause S-nitrosylation by itself but does so through the involvement of S-nitrosothiols [85].

### 3.4. Persulfidation

Persulfidation has been proposed to derive from the interaction of H_2_S with cysteine sulfenic acid formed by H_2_O_2_ and to prevent further oxidation to sulfinic acids (RSO_3_H) [86]. Persulfides can then return to thiols through the action of the thioredoxin system. Recently, Aroca et al. proposed that signaling by H_2_S occurs by protein persulfidation through the covalent addition of thiol groups to form persulfides (R-SSHs) [87,88]. Their studies revealed that protein persulfidation can be reversed by the reducing agent dithiothreitol in vitro and can thus serve to modulate protein activities and enzymes involved in the maintenance of ROS homeostasis and redox balance [88]. More recently, Shen et al. reported on how ABA stimulates the persulfidation of L-cysteine desulfhydrase 1 (DES), an endogenous H_2_S-metabolizing enzyme. In their findings, DES was activated by ABA through auto-persulfidation at Cys44 and Cys205, which led to the transient overproduction of H_2_S in an Arabidopsis guard cell. A sustainable level of H_2_S accumulation triggered persulfidation of NADPH oxidase respiratory burst oxidase homolog protein D (RBOHD) at Cys825 and Cys892 to potentiate ROS generation. Consequently, these processes initiate a negative feedback loop that helps to fine-tune guard-cell redox homeostasis and ABA signaling [89].

## 4. Details of Protein Carbonylation

### 4.1. Direct and Indirect Reactions of Protein Carbonylation

**Figure 3:**
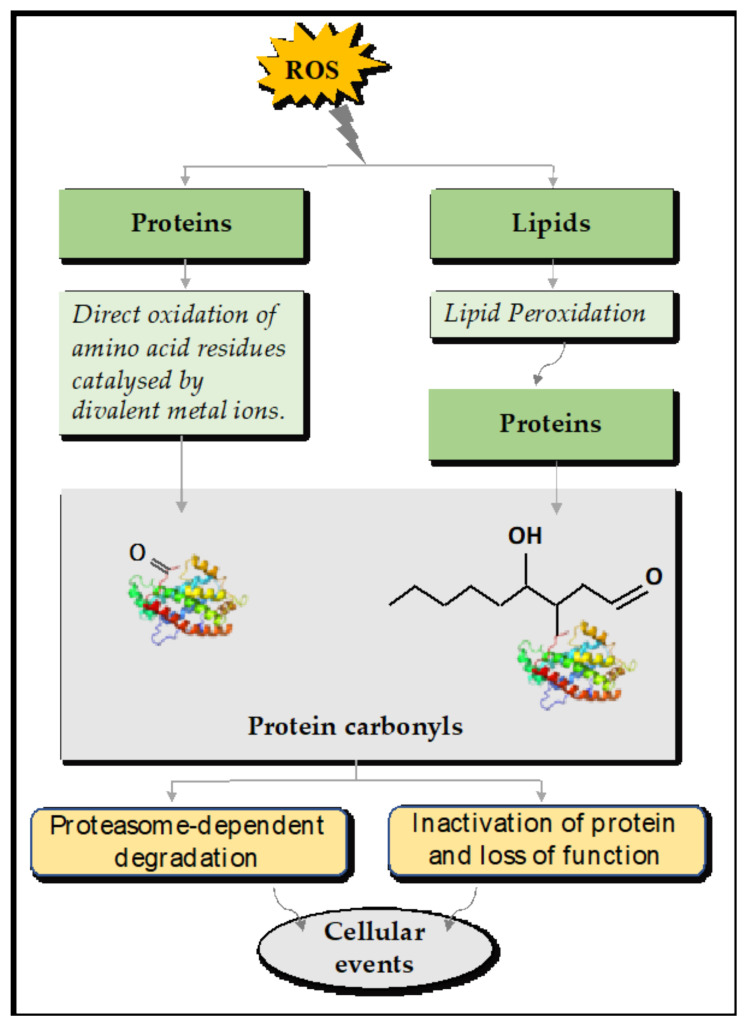
The reactions of protein carbonylation and the fates of carbonylated proteins. Metal-catalyzed oxidation of the side chains of Arg, Thr, Pro, and Lys and addition of lipid peroxidation-derived RCS to the side chains of Cys, His, and Lys represent the two types of protein carbonylation in plants and result in carbonylated proteins. The fates of carbonylated proteins. The carbonylated proteins may lose their activity, change their initial conformation, or aggregate. They are subsequently degraded by the 20S proteasome system. The biological relevance of these changes is further discussed in the text.

Protein carbonylation is an irreversible PTM that involves the introduction of aldehyde and ketone carbonyl groups into the side chains of certain amino acids [90]. It represents the most frequent and irreversible chemical modification that affects protein [91]. The chemistry of protein carbonylation is complex due to the different biomolecules that are involved, including lipid and sugar derivatives. Wong et al. proposed two major types of protein carbonylation, primary protein carbonylation and secondary protein carbonylation, to reflect how the modification occurs [92] (Figure 3). Primary protein carbonylation involves metal-catalyzed oxidation (MCO) of the side chains of Lys, Pro, and Thr residues, leading to aldehyde or ketone formation [93,94]. Primary protein carbonylation may also occur, although to a much lesser extent, through α-amidation pathways or glutamyl side chains, which leads to the synthesis of peptides with the α-keto derivative at the N-terminal [13,95]. Metal-catalyzed oxidation is one common mechanism of protein carbonylation in a biological cell [96,97,98]. It is triggered by HO^•^ derived from the Fenton reaction between Fe^2+^ (or divalent metal ions) and H_2_O_2_ in any part of the cell [99] (Figure 1B). The hydroxyl radical reacts with side chains of Lys, Pro, Arg, Thr, and sometimes Trp to cleave them and form carbonyl groups. This results in the formation of aminoadipic semialdehyde from Lys, glutamic semialdehyde from Pro and Arg, and 2-amino-3-ketobutyric acid from Thr, respectively [100].

Secondary protein carbonylation involves the addition of reactive carbonyl species (RCS) to the side chains of Cys, His, and Lys (Figure 3). RCS are electrophilic compounds that are referred to as reactive electrophile species (RES) in the literature. They are generated by the peroxidation of membrane polyunsaturated fatty acids (PUFAs; linoleic acid, linolenic acid, and arachidonic acid), particularly in the mitochondria and chloroplasts [14,30,76]. The RCS belong to different chemical classes, as summarized in Table 1: α,β-unsaturated aldehydes (4-hydroxynonenal (4-HNE) and acrolein (ACR)), keto-aldehydes (4-oxo-nonenal), isoketals, dia-aldehydes (malondialdehydes (MDA) and glyoxal), and cyclopentanones [101]. Protein and nucleic acids are the main targets of RCS and their interaction with RCS mainly occurs through Michael adduction or Schiff-base formation [101]. Examples of RES species frequently involved in protein carbonylation include 2-propenal (acrolein), 4-HNE, and malondialdehydes (MDA) [102,103,104]. A large number of carbonylated proteins have been detected and quantified in plants and non-plant species using different techniques (see below) [92,104,105,106,107].

Overall, increased protein carbonylation is associated with an increase in the cellular ROS levels under stress conditions, making carbonylated proteins a good indicator of cellular oxidative stress. It is noteworthy that carbonylated proteins are also found in unstressed cells under optimal growth conditions. This points to the involvement of protein carbonylation in cell physiological processes required for growth and maintenance. Proteomic studies have revealed that protein carbonylation is not uniform across the proteome and some proteins are more sensitive than others to carbonylation [5,108]. The pattern of protein carbonylation in *Arabidopsis thaliana* (the flowering plant) differs from that in non-photosynthetic eukaryotes. Carbonylation of proteins first increases with age (the same as other species) but declines drastically before the transition from the vegetative to the reproductive phase and independently of senescence [5]. This contrasts with the situation in animals, where increased protein carbonyls are observed with ageing. These observations indicate that protein carbonylation may serve different purposes in plants and animals.

### 4.2. The Fates of Carbonylated Proteins

Carbonylation by RCS increases the hydrophobicity of proteins, which often become partially unfolded. As a consequence, protein carbonylation usually deactivates the protein function [15]. Studies have shown that carbonylated proteins are degraded by the proteasome system [15,116,117] (Figure 3). Hence, carbonylated proteins were deemed a marker for proteolysis. In contrast to ubiquitinylated proteins that are degraded by the 26S proteasome system, the degradation of carbonylated proteins only requires the 20S proteasome system [118]. It should be noted that the steady-state level of carbonylated proteins depends not only on their formation but also on their degradation. Therefore, increased carbonyl levels may also stem from a dysfunctional proteasome system. When proteins are heavily carbonylated, they tend to accumulate as cytotoxic aggregates due to their increased hydrophobicity. Such aggregates are linked to age-related diseases in humans and to the clogging of the proteasome system [13].

The turnover of the cellular proteome is also mediated by autophagy, and autophagy mutants displayed delayed growth compared to the wild type [119,120,121]. Interestingly, RNAi-AtATG18a transgenic lines of Arabidopsis that were impaired in autophagy were found to accumulate significant levels of carbonylated proteins compared to the wild type [122,123]. These findings indicate that autophagy also serves to degrade carbonylated proteins in plants, but the mechanism that recognizes and directs the carbonylated proteins to the autophagosome remains to be elucidated. In yeast and mammals, p62 and NBR1 (neighbor of BRCA1) act as cargo receptors to target protein aggregates derived from extensive ubiquitination in order to achieve degradation by selective autophagy [124]. A hybrid protein of p62 and NBR1 has also been identified, which mediates ubiquitin-dependent selective autophagy in plants [125,126]. It is still unclear whether protein carbonylation represents a posttranslational modification sufficient to target proteins to the autophagosome. If not, the cargo receptor that recognizes carbonylated proteins remains to be uncovered. This would improve our understanding of the relevance of protein carbonylation under normal growth conditions.

## 5. Importance of Protein Carbonylation in Seed After-Ripening and Germination

Fresh seeds of most temperate species are dormant and will not germinate at harvest. Seed after-ripening refers to the period in which seeds are air-dried and stored. It is associated with dormancy release and allows synchronized and faster seed germination later, although seed after-ripening and loss of dormancy have been shown to be two distinct physiological processes [127,128]. Dormancy release by environmental cues allows seed germination. Though many proteins were carbonylated in *Arabidopsis thaliana* seeds during germination, seeds successfully grew into highly vigorous seedlings and young plants, thus indicating a role for protein carbonylation in seed dormancy release and germination [11]. Protein carbonylation could be involved in the transitions from seed dormancy to seed germination and ageing [6]. Parallel to ROS increase, protein carbonylation during seed storage was shown to alleviate dormancy in sunflower and Arabidopsis [129,130]. Reactive oxygen species produced by the NADPH oxidase AtrbohB in Arabidopsis during after-ripening induced protein carbonylation events in the seeds of the Brassicaceae model species *Lepidium sativum* and *A. thaliana* [131]. Consistently, the mutation of the NADPH oxidase genes *AtRbohB* and *AtRbohD* in Arabidopsis reduced protein carbonylation and increased seed dormancy [131]. Furthermore, natural ageing of seeds or controlled heating of seeds to mimic natural seed ageing led to abundant carbonylated proteins and complete loss of germination in rice and Arabidopsis [132]. This suggests a balance between the beneficial effect of protein carbonylation in breaking seed dormancy and the adverse effect on seed viability. It remains unclear how such a balance is achieved and what determines the specificity of protein carbonylation during seed after-ripening and germination.

## 6. Importance of Protein Carbonylation in Proteome Remodeling under Nutrient Starvation and Stress Conditions

Protein degradation represents a key cellular process that assures a healthy proteome and helps recycle amino acids during nutrient starvation or stress. As we know, the level of carbonylated proteins rises in stressed cells. Prolonged abiotic stress often results in nutrient starvation, the stress in plants causing a significant overlap between stress-induced genes and sugar starvation-responsive genes [133,134]. Extended dark-induced sucrose starvation is associated with an increase of genes involved in proteolysis [135,136,137,138]. The increased level of carbonylated proteins under stress, therefore, coincides with a rise of proteolysis, which provides cells with amino acids for respiration. This suggests that protein carbonylation could be relevant to proteolysis associated with cell growth and maintenance, particularly under stress. Indeed, nutrient starvation resulted in increased protein carbonylation in a sub-population of an *E. coli* culture [139,140]. *E. coli* cells deficient in proteolysis accumulated a high level of carbonylated proteins, indicating that carbonylated proteins are targeted for proteolysis [141]. Similarly, serum starvation of two cancer cell lines, A549 and PC3, was found to increase the levels of carbonylated proteins revealed by a benzocoumarin hydrazine in vivo labeling probe [142]. Carbon starvation was also found to increase protein carbonylation and the activity of the 20S proteasome in maize root tips [143]. Hence, like ubiquitination, protein carbonylation appears as an effective way of remodeling the cellular proteome, particularly under stress conditions that challenge energy metabolism. With regard to this, oxidative stress often causes the carbonylation and deactivation of glycolytic and citric acid cycle enzymes and, accordingly, bacteria and animal cells respond to oxidative stress with about a 50% decrease in intracellular ATP levels [144,145]. Under this condition, cells must rely on ATP-independent processes to counteract stress and recycle oxidized proteins. Low intracellular ATP levels were found to decrease the 26S proteasome levels but to increase 20S proteasome levels [146]. In contrast to ubiquitylation-dependent degradation by the 26S proteasome, protein carbonylation does not require ATP and enzymes. The degradation of carbonylated proteins by the 20S proteasome system constitutes an energy-efficient way to quickly provide amino acid building blocks for growth [96,147,148].

Protein carbonylation is traditionally deemed damaging to cells, but recent studies in mammals and bacteria indicate that the oxidation of certain proteins turns them into chaperones, which direct other proteins to the 20S proteasome system [148]. Several proteins, including members of the heat shock protein 70 families, were found to turn into ATP-independent chaperones that refold proteins or direct them to the proteasome system [148]. The chaperone Hsp70 was shown to be essential for the stabilization of the 19S particle and the reassembly of the 26S proteasome system [117], whereas low levels of Hsp90 led to the disassembly of the 26S proteasome and the increase of the 20S particles [149]. Both the Hsp70 and HSP90 proteins are frequently identified as being carbonylated in bacteria, animal, and plant species [150]. Their carbonylation results in the destabilization of the 26S proteasome and increased 20S proteasome particles that are required for degrading oxidized proteome [118,143]. The 26S proteasome is heavily dependent on ATP, whereas the 20S is not but becomes particularly essential for the cells under stress conditions [151]. An impairment of the 26S proteasome system has been associated with an increase of 20S particle levels in plants [152]. Arabidopsis plants deficient in the 26S proteasome system showed increased activity of the 20S system and were more resistant to treatments that promote protein oxidation [152]. An increase in the 20S proteasome contributed to enhancing oxidative stress tolerance in plants [153]. This antagonistic regulation of the 26S and 20S proteasome across bacteria, animals, and plants likely assures the maintenance of a healthy proteome under both normal and stress conditions. Overall, the rise of protein carbonylation might serve to maintain protein turnover in a context of low ATP and contribute to oxidative stress tolerance. This is particularly relevant in scenarios of short-term stress where the concomitant expression of ROS- and RCS-detoxification enzymes quickly help the plant overcome the stress.

## 7. Protein Carbonylation Serves as a Signal Transduction Mechanism in Bacteria and Mammalian Cells

Signal transduction is a process that converts one form of a signal into another type within cells. ROS serve as the second messenger for signal transduction processes; however, their molecular targets have not been fully identified [76]. Studies in prokaryotes and humans have demonstrated the involvement of protein carbonylation as an ROS signal transduction mechanism. To illustrate our point, we have chosen to describe only a few examples before focusing on current evidence in plants.

### 7.1. Carbonylation of the Transcription Repressor PerR Facilitates H_2_O_2_ Sensing and the Expression of Oxidative Response Genes in Prokaryotes

**Figure 4:**
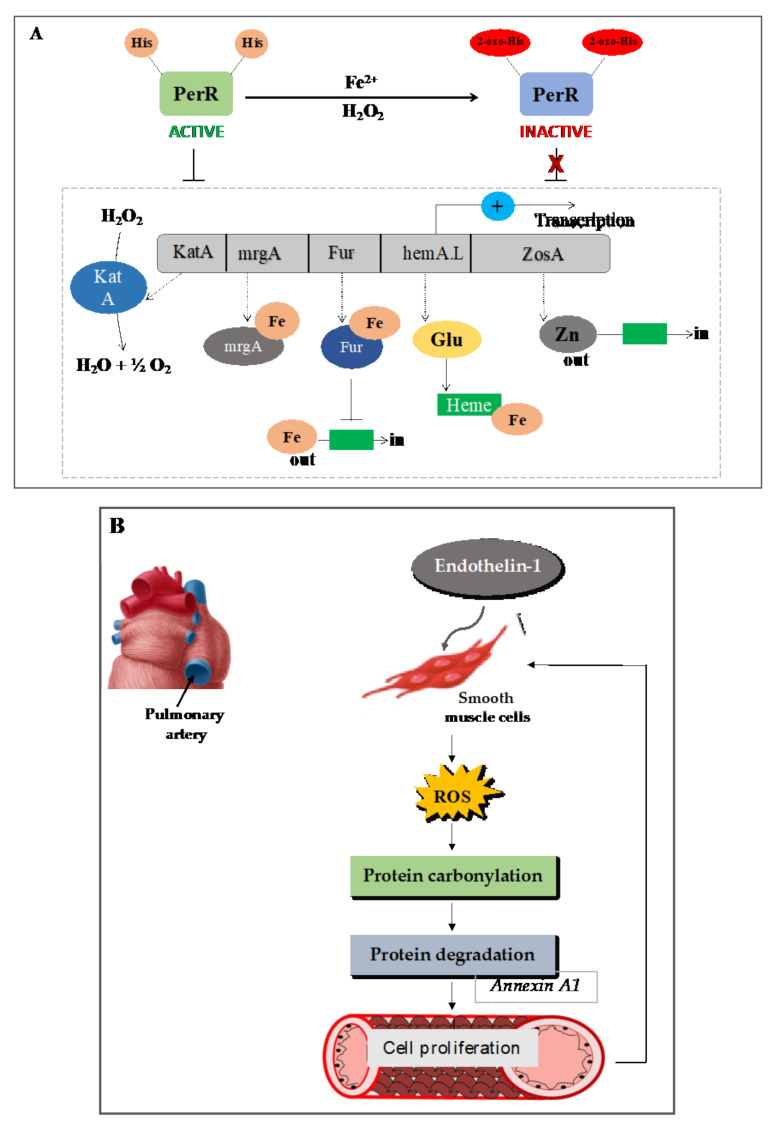
Examples of the implication of protein carbonylation in ROS signaling in prokaryotes and humans. (**A**) *Bacilius subtilis* uses metal-catalyzed oxidation of PerR for H_2_O_2_ and peroxides sensing. The transcription factor PerR possesses two His residues that bind to Fe. Exposure of PerR to a low level of H_2_O_2_ is sufficient to trigger one or both His residues’ oxidation, presumably via the Fenton reaction that involves the bound iron. This causes the loss of the DNA binding activity of PerR and the derepression of the PerR regulon-encoding enzymes, such as the genes *katA* (catalase), *mrgA* (nonspecific DNA-binding protein), *hemAXCDBL* (heme biosynthesis operon), *Fur* (iron uptake regulator), and ZosA (zinc-transporting ATPase), acting to detoxifying peroxides. The induced *katA* (catalase) removes the hydrogen peroxide to water, whereas Fur suppresses the iron intake. (**B**) Mammalian annexin A1 undergoes carbonylation as a signal transduction mechanism in response to endothelin-1 (ET-1) treatment to promote cell proliferation and apoptosis. ET-1 is known as a potent vasoconstrictor and a mitogen of pulmonary artery smooth muscle cells. ET-1 activates the proliferation of pulmonary artery smooth muscle cells through the production of ROS. ROS trigger metal-catalyzed oxidation of annexin A1 in response to ET-1 treatment. The carbonylation of annexin A1 and its subsequent degradation of promote cell proliferation and survival. The figures are based on data from the literature [154,157,158,159,160,162,163,164,168,169,170,171,172,173].

Bacteria adapt to an elevated level of ROS by increasing the expression of detoxifying enzymes and repair proteins. The main ROS detoxification enzymes in bacteria include catalases, catalase/peroxidases, alkyl hydroperoxide reductase (AhpR), peroxiredoxins, superoxide dismutases, and the organic hydroperoxide resistance protein (Ohr) [154,155,156,157]. In *B. subtilis*, iron derepresses oxidative stress genes via PerR, a transcription factor related to the ferric-uptake repressor (Fur) family of the metalloproteins [158]. PerR was shown to mediate H_2_O_2_- and metal-dependent induction of the genes katA (catalase), ahpCF (alkyl hydroperoxide reductase), mrgA (nonspecific DNA-binding protein), and hemAXCDBL (heme biosynthesis operon) [159,160]. The mechanism of H_2_O_2_ sensing is thought to be mediated by a typical protein thiol (redox-active cysteine) [154,161,162,163]. Indeed, several transcription factors have been identified in bacteria, yeast, and mammals, which use the reversible oxidation of cysteine to sense H_2_O_2_ [76,124,162]. However, Lee and Helmann have revealed that H_2_O_2_ sensing occurs by metal-catalyzed oxidation of PerR and leads to the expression of oxidative defense genes (Figure 4A). PerR is a zinc-binding protein with a regulatory site that coordinates either Fe^2+^ or Mn^2+^ metal ions. There are two His residues present in the transcription factor PerR of *B. subtilis*, which coordinate with Fe. Upon exposure of PerR to a low level of H_2_O_2_ (˂10 µM), one or both His residues become oxidized, presumably by the hydroxyl radical generated by the Fenton reaction involving the bound iron [164]. This causes the loss of the DNA binding activity of PerR and the derepression of the PerR regulon-encoding enzymes katA, ahpCF, mrgA, and hemAXCDBL (heme biosynthesis operon) [159,160]. Hence, in the presence of iron (Fe^2+^), PerR mediates strong induction of the PerR regulon in response to H_2_O_2_ (Figure 4A). PerR represents the major regulator of the peroxide-induced stress response in both Gram-positive and Gram-negative bacteria and its carbonylation constitutes a widespread mechanism of ROS and peroxide sensing in the procaryotes [162].

### 7.2. In Animals: Mammalian Cell

Numerous cases in which protein carbonylation mediates ROS signaling have been reported in animal cells [165]. Endothelin-1 (ET-1) is a potent vasoconstrictor and a mitogen of smooth muscle cells of the pulmonary artery [166,167,168]. ET-1 is activated by receptors ETA or ETB, which can induce the proliferation of pulmonary artery smooth muscle cells through the production of ROS [169,170,171]. In an animal model of pulmonary hypertension, the expression of endothelin-1 was high and further progression of the disease was blocked by endothelin-1 receptor antagonists [172,173]. ET-1 induces ROS through NAD(P)H oxidase, and antioxidants were found to block endothelin-1-induced proliferation of the smooth muscle cells in the fetal bovine pulmonary artery [169,170]. The idea that protein carbonylation might play a role in ET-1 signaling arose is based upon observations that low concentrations of H_2_O_2_ (500 nM) could induce protein carbonylation and that ET-1 triggered protein carbonyl as early as 5–10 minutes in cultured bovine pulmonary artery smooth muscle cells [168,174]. When pulmonary artery smooth muscle cells were pre-treated with ET-1 receptor antagonists, hydrogen peroxide scavengers, or an iron chelator (deferoxamine), subsequent treatment with ET-1 was found to promote protein carbonylation in an ET-1-receptor- and Fenton reaction-dependent manner [168,174]. Annexin A1 was identified as one prominently carbonylated protein in response to ET-1 using 2D-PAGE and mass spectrometry. Annexin A1 inhibits cell growth and promotes apoptosis but its carbonylation and subsequent degradation in response to ET-1 led to cell proliferation [174] (Figure 4B). These findings demonstrate that metal-catalyzed protein carbonylation could be promoted in response to ligand–receptor interactions.

Similarly, the regulation of phase II antioxidant enzyme expression by the nuclear factor (erythroid-derived 2)-like 2 (NRF2)–Kelch-like ECH-associated 1 (KEAP1) pathway involves protein carbonylation [175]. KEAP1 is a Cullin3 ubiquitin ligase complex adaptor protein. Under physiological conditions, KEAP1 binds to NRF2 in the cytoplasm and sequesters it from the nucleus by targeting it for degradation by the proteasome system. In response to oxidative stress, KEAP1 is modified with HNE (reactive carbonyl species) through carbonylation at critical cysteine residues (Cys273 and Cys288); this releases NRF2 that translocates into the nucleus where it forms a dimer with a variety of nuclear factors, including MAF and NRF1 [176]. This complex binds to the antioxidant response element (ARE), which triggers the expression of the antioxidant responsive genes. Fang and Holmgren also reported that when thioredoxin (TRX) became carbonylated with HNE, this modified the structure of TRX at the vicinal thiol groups of TRX (Cys32 and Cys35) and triggered the release of apoptosis signaling-regulatory kinase 1 (ASK1) from the complex in which it was sequestered. This facilitates its autophosphorylation at Thr813, Thr838, and Thr842 and subsequent activation [177]. Additionally, TRX can be carbonylated at Cys72, a residue distal to the catalytic site resulting in TRX inactivation, but this did not trigger the release of ASK1. Activation of ASK1 triggers a cascade of phosphorylation of SEK and c-Jun N-terminal kinase (JNK), leading to nuclear factor-kβ activation [178] and the development of insulin resistance [165,179,180]. These signaling events illustrate a negative feedback loop in which lipid peroxidation-induced protein carbonylation transduces the ROS signal to allow the expression of antioxidant response genes and phase II metabolic enzymes in response to oxidative stress.

## 8. The Implication of Protein Carbonylation in Phytohormone Signaling Pathways

The phytohormone abscisic acid (ABA) is known to induce the production of ROS by NAD(P)H oxidases AtrbohD and AtrbohF located on the plasma membrane in the Arabidopsis guard cells. ROS-induced lipid peroxidation products, including MDA and HNE, are commonly identified as RES or RCS as a result of the presence of an electrophilic α,β-unsaturated carbonyl group [14,30]. Islam et al. demonstrated that RCS function downstream of H_2_O_2_ production in ABA signaling for stomatal closure in guard cells using transgenic tobacco plants overexpressing 2-alkenal reductase [18]. In the epidermal tissue treated with ABA, RCS levels increased within 30 min and remained high up to 120 min. Both ABA and H_2_O_2_ induced the production of RCS [18]. Treatment of the epidermal tissues with either 50 µM ABA or 1 mM of H_2_O_2_ significantly increased the level of acrolein and HNE content. The application of the RCS scavengers carnosine (1 mM) and pyridoxamine (0.5 mM) did not affect the ABA-induced H_2_O_2_ production but inhibited the ABA and H_2_O_2_-induced stomatal closure [181].

Similar to ABA-induced stomatal closure, the production of ROS by NAD(P)H oxidases is also required by MeJA-induced stomatal closure in A. thaliana guard cells [182,183,184,185,186]. As with ABA, the application of MeJA induced the accumulation of RCS such as acrolein and HNE in WT tobacco plants, thus implicating RCS in MeJA-induced stomatal closure [19]. A study by Akter and colleagues revealed that stomatal closure induced by MeJA is accompanied by depletion in the level of intracellular GSH found in Arabidopsis guard cells [187], but no effect of GSH was found in MeJA-induced ROS production [188]. GSH is thus required downstream of ROS. Consistently, RCS production triggered by MeJA induced GSH depletion in guard cells [19], indicating that RCS targets likely lie downstream of H_2_O_2_ production by RbohD and RbohF proteins in the guard cells.

**Figure 5:**
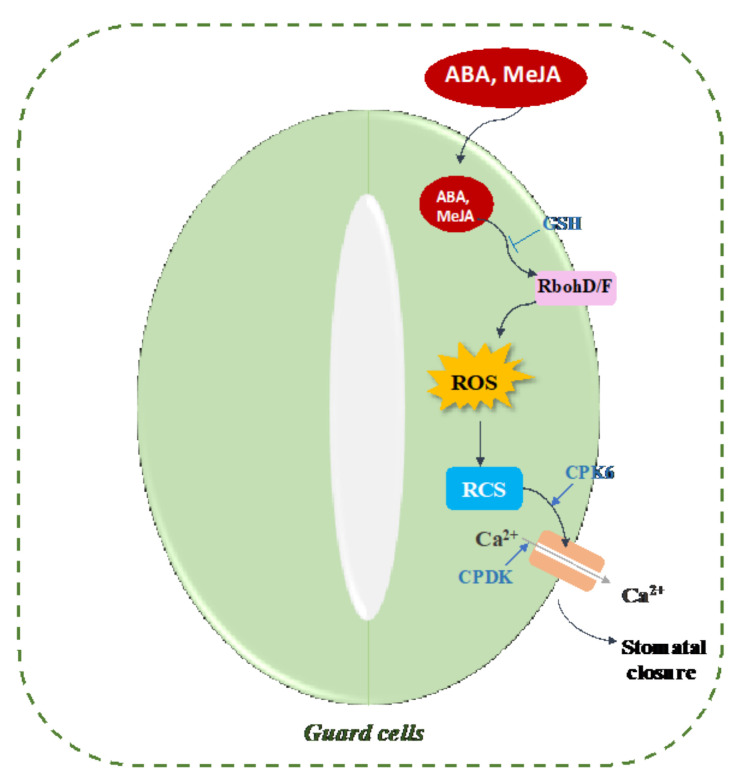
A model of RCS-mediated carbonylation processes in the hormonal signaling pathway in guard cells. ABA or MeJA induced the production of ROS by NAD(P)H oxidases (RbohD and RbohF) located on the plasma membrane in the guard cells. The RCS derived from the oxidation of membrane lipids by ROS trigger the activation of Ca^2+^-permeable cation channels in the plasma membrane, which initiates the elevation of the cytosolic free Ca^2+^ concentration through Ca^2+^ influx from the apoplast and the intracellular stores. RCS likely induce the carbonylation of an unknown protein target upstream of the calcium channels. CPK6 is a positive regulator of ABA- or MeJA-induced stomatal closure, whereas CDPK6, an isozyme of CPK6, is responsible for the regulation of Ica channels in ABA- or MeJA-induced stomatal closure. The increase in cytosolic Ca^2+^ is sensed by CDPKs and CPK6 and results in the activation of the S-type anion channels and stomatal closure. GSH is a negative regulator of ABA or MeJA signaling in the guard cell-induced stomatal closure by depletion of intracellular levels of GSH. This model is based on the previous studies from the literature [18,19,181,187,188,190,191,193].

The calcium ion plays a key role as a second messenger in ABA signaling and ABA-induced stomatal closure in the guard cell [189,190,191]. An elevation of cytosolic free Ca^2+^ concentration ([Ca^2+^]cyt) was also associated with methyl jasmonate (MeJA)-induced stomatal closure in guard cells [186]. The activation of Ca^2+^ permeable cation channels in the plasma membrane triggers the elevation of the cytosolic free Ca^2+^ concentration through Ca^2+^ influx from the apoplast and the intracellular stores [182,183,192]. H_2_O_2_ activates Ca^2+^-permeable cation (Ica) channels to trigger the elevation of the [Ca^2+^]cyt level in the guard cells [182]. The activation of the Ica channels was enhanced in the GSH-depleted mutant *cad2–1* [193], indicating that GSH negatively controls H_2_O_2_-mediated Ica channel activation in Arabidopsis. Exogenous application of the RCS acrolein was more effective at raising the level of [Ca^2+^]cyt and inducing stomatal closure than exogenous H_2_O_2_ application [181]. Similarly, RCS-mediated [Ca^2+^]cyt increase was negatively regulated by GSH [19]. These suggest that GSH acts downstream of H_2_O_2_ and RCS most likely by scavenging RCS to prevent them from reacting with protein targets. Collectively, the findings generated over the last decade by Mano’s group position RCS as signal mediators downstream of H_2_O_2_ production by RBOHs and upstream of the elicitation of the Ca^2+^ signal in Arabidopsis guard cells for both ABA- and MeJA-mediated stomatal closure (Figure 5). Treatment of guard cells with 100 µM acrolein and HNE induced stomata closure incrementally for 4 h. Interestingly, the stomata closure could be reversed when replacing the bathing solution with one without acrolein or HNE [181]. These observations further point to an increased turnover rate of the proteins targeted by RCS, given that protein carbonylation is irreversible.

ROS are also known to control several developmental processes, including leaf expansion and xylem differentiation, adventitious root formation, and root hair development [194,195,196,197]. The involvement of ROS specifically in lateral root (LR) formation was reported in soybean [198], rice [199], and *Phaseolus vulgaris* [200]. In these studies, H_2_O_2_ was found to accumulate at the initiation sites where the lateral root emerged from. Several reports suggested that the LR formation might be modulated by the interaction between auxin and ROS signals, with auxin stimulus inducing the production of ROS through the activation of RBOHs in the LR-forming regions. Treatment of Arabidopsis roots with auxin triggered the accumulation of H_2_O_2_ through RBOHs and promoted lateral root formation, whereas RBOH-deficient mutants produce fewer LRs than the wild type in Arabidopsis [172,201,202,203,204]. Despite these findings, the mechanism of action of ROS in auxin signaling for LR emergence was still unclear until recently. By investigating this mechanism, Biswas and colleagues established that RCS derived from ROS mediate auxin signaling to promote lateral root formation [16]. The levels of RCS, including acrolein, HNE, and crotonaldehyde, were elevated before the formation of LRs in Arabidopsis following auxin treatment, and supplementation of the carbonyl scavenger carnosine suppressed auxin-induced LR formation (both in numbers and density) [16]. The action of the RCS to promote LR formation depended on the presence of auxin receptors (TIR1 or AFB2) and promoted the degradation of Aux/IAA proteins. RCS likely further auxin signaling by triggering the degradation of negative regulators of the auxin signaling pathways via protein carbonylation. A summary of the recent findings pointing to hormone signal transduction by protein carbonylation in plants is provided in Table 2.

## 9. Crosstalk Between Carbonylation and Other PTMs

Cys residues are primary targets for ROS-mediated PTMs. As described above, Cys residues can undergo nitrosylation, glutathionylation, persulfidation, and direct oxidation to sulfenic, sulfinic, or sulfonic acid derivatives. These PTMs on Cys are associated with diverse biological effects [2]. Since Cys is also a substrate for carbonylation, it is very likely that carbonylation interferes positively or negatively with these biological effects, particularly at the onset of stress and in the early events of stress signaling in plants and non-plant species. The carbonylation of Keap1 by RCS alters the adaptor function of Keap1 and prevents the ubiquitination of its specific partner NRF2 [123,205]. Mitogen-activated protein kinases (MAPKs) form an important group of proteins that relay intracellular and extracellular signals via a cascade of protein phosphorylation in eukaryotic cells. Reactive carbonyl species were found to modify MAPKs to interfere with their signaling functions [206,207]. Crosstalk between protein carbonylation and protein nitrosylation has also been reported [6]. The analysis of the proteome of citrus plants (*Citrus aurantium* L.) exposed to salt stress revealed an important overlap between the carbonylated proteins and the nitrosylated proteins after pretreatment with H_2_O_2_ and sodium nitroprusside, respectively [208]. Sodium nitroprusside is a donor of ˙NO required for protein nitrosylation. Interestingly, H_2_O_2_ and SNP pre-treatments before salinity stress lowered the levels of both carbonylated proteins and S-nitrosylated proteins, indicating crosstalk between H_2_O_2_ and ˙NO signaling pathways [208,209]. The irreversible carbonylation of proteins may thus prevent reversible PTMs, such as S-nitrosylation and phosphorylation, from occurring or vice versa. This is supported by observations in animals and the effects of NO and HCN in alleviating protein oxidation in seeds [11,129,180,210,211]. Besides Cys, carbonylation at Lys residues influences the effects of acetylation, methylation, mono- and polyubiquitination, and SUMOylation of proteins. Histones are lysine- and arginine-rich proteins that regulate chromatin structure and gene expression. Treatment of RKO cells with either 4-HNE or 4-oxo-2-nonenal resulted in the carbonylation of histones at Lys and His residues [212]. Complementary tests in vitro revealed that pre-treatment of H3/H4 tetramers inhibited nucleosome assembly similarly to lysine acetylation. Combined histone acetylation and carbonylation may thus enhance gene activation in the cell. So far, crosstalk between histone protein carbonylation and histone acetylation or phosphorylation has yet to be demonstrated in plants. Progress brought about by studies in animals show that much remains to be known in plants concerning the importance of protein carbonylation in ROS, hormone signaling, and their crosstalk.

## 10. Target Specificity in Protein Carbonylation

The answer to the question concerning specificity in protein carbonylation has long been sought. Despite being nonenzymatic, protein carbonylation appears to bear some substrate specificity according to the hundreds of studies in several prokaryote and eukaryote species [150]. Enzymes are the most frequent targets identified in various species, probably because of the enhanced nucleophilic reactivity of the residues in their active sites, namely Cys, His, and Lys. Besides enzymes, heat shock proteins and cytoskeleton proteins are often identified among carbonylated proteins [150,213]. Several orthologous or conserved enzymes and proteins have been found to be carbonylated similarly across species and kingdoms. A long list of such enzymes and proteins found in *Escherichia coli*, rats, humans, and plants has been drawn up and constitutes a solid case in favor of the specificity of protein carbonylation [150]. However, how can one predict such specificity? From the analysis of carbonylated *E. coli* proteins and bovine serum albumin (BSA) by mass spectrometry, Maisonneuve et al. identified small peptide regions called RKPT-enriched regions containing several carbonylated residues. Based on these regions and surrounding residues, they developed a computer model (available at http://www.lcb.cnrs-mrs.fr/CSPD/, accessed on 27 May 2021) capable of predicting sites and proteins more susceptible to carbonylation in *E. coli* [97]. The model, however, could only predict direct carbonylation products in *E. coli* and failed to detect carbonylated proteins found in *A. thaliana* and yeast. To overcome these limitations, another bioinformatics tool, named CarSPred, was developed later to predict carbonylation in the human proteome [214]. Interestingly, these studies and several others agreed upon the fact that RKPT-enriched regions in proteins are hot spots of protein carbonylation in several species [214,215,216,217]. A similar or better bioinformatics tool is still lacking for plants. An important step toward closing this gap is to further improve carbonylated proteome sequencing and develop new approaches to identify modified residues unequivocally (see below).

## 11. Challenges and Approaches for Studying the Roles of Protein Carbonylation in Plants: Lessons from Studies in Mammalians

Protein carbonylation is irreversible in most cases and carbonylated proteins can remain in the cell for more than 4 h before degradation [218]. Several techniques have been developed over the last two decades to analyze the profile of protein carbonylation or to identify the carbonylated proteins within the cellular proteome. These methods range from Western blot analyses to mass spectrometry-based protein sequencing. For a Western blot analysis, carbonylated proteins are first labeled with a carbonyl-reactive compound, most commonly 2,4-dinitrophenylhydrazine (DNPH), prior to the separation by electrophoresis and then revealed using anti-DNP antibodies. DNPH reacts with the aldehyde or ketone carbonyl group and forms hydrazone derivatives (DNP), thus enabling spectrophotometric or antibody detection [219,220].

The aldehyde reactive probe (ARP; N′-aminooxymethylcarbonylhydrazino D- biotin), a biotinylated hydroxylamine compound that forms an oxime derivative with the carbonyl group found in oxidatively modified proteins, is also frequently used [221,222]. A comprehensive description of the existing methods has been undertaken in previous studies [150,223]. For mass spectrometry analysis, an enrichment step of carbonylated proteins is often required, as for most PTM analyses [17,93,108,224,225,226,227]. A major drawback of this approach though is the impossibility of revealing the sites of the modification and quantifying the number of carbonylated proteins, particularly when the modified peptide is not found and sequenced. Moreover, diverse RCS are generated in the cell, the majority of which are unknown, and therefore the exact mass adducts brought by the RCS cannot be determined precisely and accounted for in the mass search analysis. Although a few hits can be found based on the known mass of the commonly found RCS in the cells (HNE, MDA, acrolein), this approach still under-samples the carbonylated proteome. This is as well as the fact that the mere identification of the carbonylated proteins does not suffice to conclude about the biological effect of the modification in vivo. However, because of the enrichment step during the sample preparation, the approach still provides a list of putative carbonylated proteins that can be further examined by top-down proteomics. Pioneer and recent studies in mammal and plant cells adopted this strategy and were successful in validating the biological effects of the carbonylation of candidate targets identified in a first screening [225,228,229,230]. A more robust strategy to identify the site of modification consists of profiling the carbonylated proteins based on their affinity to a given RCS. This approach, called affinity-based proteome profiling (ABBP), has been successfully used in mammal cells to identify cysteines that are highly reactive to RCS [231,232,233]. Genuine targets of protein carbonylation in vivo have been identified using this method and the biological effects of the modification in gene signaling were successfully characterized thereafter by site-directed mutagenesis and mutant analysis [231,232]. Probes that are blind to the nature of the target amino acid residues have also been developed to identify residues other than Cys [234]. We are currently unaware of the use of these probes with plant samples. Furthermore, one critical research challenge relates to the confounding feature of protein carbonylation—in other words, how can we distinguish potential physiological effects of protein carbonylation (protein quality control and recycling, crosstalk, and signaling) from the fatality of severe oxidative stress where an unavoidable surge of protein carbonylation leads to protein aggregation and cell death? A targeted proteomics approach could help. More knowledge needs to be accumulated on single proteins to build up a global understanding of the effects of their carbonylation on the biological functions known for them. Another powerful approach that allows the probing of protein carbonylation in vivo has been developed for mammals [215,235]. Due to the proven efficacy and robustness of these chemical and genetics tools, they could also be used in plants to bring progress in the field.

## 12. Conclusion and Future Perspectives

Redox biology has gained much attention in plant science over the last two decades. The traditional view of ROS as deleterious compounds has evolved into the acknowledgment of the importance of ROS in plant physiology, growth, and development [236,237,238]. ROS-mediated PTMs have emerged as signal transduction mechanisms that relay environmental stress and hormone cues toward and within the cell. Of these PTMs, the importance of protein carbonylation in redox biology has begun to surface through pioneering studies in plants and the recent findings on its implication in ABA, auxin, and JA signaling pathways [13,76,239,240,241,242,243]. The proteins targeted by carbonylation for the transduction of these hormone signals have yet to be identified. In contrast to protein ubiquitination and phosphorylation, it is still unknown how protein carbonylation is controlled and what other physiological processes are related to it. Moreover, diverse RCS are produced in the cell and likely have different effects depending on their chemical nature and the proteins that they modify [244]. As a consequence, the identification of the RCS responsible for the modification in vivo remains challenging, as the timing, the location, and the nature of the RCS mediating the modification can vary. However, a combined approach involving screening for reactivity using arbitrary RCS substrates followed by targeted characterization, as well as the use of ion mobility spectrometry, may be helpful [105,234]. For signaling, a target protein must have a high affinity with the RCS and be able to translate the RCS signal into a downstream protein via other modifications. For a target protein, the ratio of the carbonylated forms to the non-carbonylated form is often unclear. As pointed out by Poganik et al. [245], the carbonylation of a protein involved in ROS or hormone signaling pathways is likely to have a dominant effect even when only a small fraction of the pool of the target protein is carbonylated. We are very hopeful that the next few years will witness ground-breaking findings and answers to these questions about the importance of protein carbonylation in plant redox biology.

The analysis and summary of the findings provided in this review revealed the emerging role of protein carbonylation in protein quality control, protein homeostasis, and hormone signaling. As in prokaryotes and eukaryotes, these functions of protein carbonylation could also be explored in plant research and redox biology. The findings could then be used in biotechnology to mitigate the effects of environmental stress on crops.

## Figures and Tables

**Table 1:** Reactive carbonyl species (RCS) frequently implicated in protein carbonylation-mediated gene signaling.

Lipid Peroxide-Derived Reactive Carbonyl Species	Predominant PUFAs	Preference of Amino Acids for Modification	Type of Reaction with Amino Acids	References
4-Hydroxy-(E)-2-nonenal (4-HNE)	Linoleic acid (LA: 18:2ω-6) Arachidonic acid (AA: 20:4, ω-6)	Cys >> His > Lys	Michael addition	[108,109,110,111,112,113]
Malondialdehyde (MDA)	Arachidonic acid (AA: 20:4, ω-6)	Lys >> His > Arg	Michael addition	[7,113,114]
Acrolein	Linoleic acid (LA: 18:2ω-6)	Cys >> His > Lys	Michael addition or Schiff-base formation	[108,115]
4-Oxo-nonenal (4-ONE)	Linoleic acid (LA: 18:2ω-6) Arachidonic acid (AA: 20:4, ω-6)	Lys >> Cys > His > Arg	Schiff-base formation	[110,113]

**Table 2:** Summary of RCS-mediated protein carbonylation in plants.

Hormonal Signaling	Physiological Processes	Forms of RCS Involved	References
Auxin signaling	Lateral root formation	HNE, acrolein, crotonaldehyde, butyraldehyde	[16]
ABA signaling	Stomatal closure	HNE, MDA	[17,18,183]
MeJA signaling	Stomatal closure	HNE, MDA	[19]

## Data Availability

No new data were created or analyzed in this study. Data sharing is not applicable to this article.
